# Dysregulation of *Mycobacterium marinum* ESX-5 Secretion by Novel 1,2,4-oxadiazoles

**DOI:** 10.3390/biom13020211

**Published:** 2023-01-21

**Authors:** Vien Q. T. Ho, Mark K. Rong, Eva Habjan, Samantha D. Bommer, Thang V. Pham, Sander R. Piersma, Wilbert Bitter, Eelco Ruijter, Alexander Speer

**Affiliations:** 1 Department of Medical Microbiology and Infection Control, Amsterdam UMC, Vrije Universiteit Medical Center, 1081 HV Amsterdam, The Netherlands; 2 Department of Chemistry and Pharmaceutical Sciences, Amsterdam Institute of Molecular and Life Sciences (AIMMS), Vrije Universiteit Amsterdam, 1081 HV Amsterdam, The Netherlands; 3 Department of Medical Oncology, OncoProteomics Laboratory, AmsterdamUMC, Vrije Universiteit Medical Center, 1081 HV Amsterdam, The Netherlands; 4 Department of Molecular Microbiology, Amsterdam Institute of Molecular and Life Sciences (AIMMS), Vrije Universiteit Amsterdam, 1081 HV Amsterdam, The Netherlands

**Keywords:** type VII secretion, protein secretion, ESX-5, mycobacteria, *Mycobacterium marinum*

## Abstract

The ESX-5 secretion system is essential for the viability and virulence of slow-growing pathogenic mycobacterial species. In this study, we identified a 1,2,4-oxadiazole derivative as a putative effector of the ESX-5 secretion system. We confirmed that this 1,2,4-oxadiazole and several newly synthesized derivatives inhibited the ESX-5-dependent secretion of active lipase LipY by *Mycobacterium marinum* (*M. marinum*). Despite reduced lipase activity, we did not observe a defect in LipY secretion itself. Moreover, we found that several other ESX-5 substrates, especially the high molecular-weight PE_PGRS MMAR_5294, were even more abundantly secreted by *M. marinum* treated with several 1,2,4-oxadiazoles. Analysis of *M. marinum* grown in the presence of different oxadiazole derivatives revealed that the secretion of LipY and the induction of PE_PGRS secretion were, in fact, two independent phenotypes, as we were able to identify structural features in the compounds that specifically induced only one of these phenotypes. Whereas the three most potent 1,2,4-oxadiazoles displayed only a mild effect on the growth of *M. marinum* or *M. tuberculosis* in culture, these compounds significantly reduced bacterial burden in *M. marinum*-infected zebrafish models. In conclusion, we report a 1,2,4-oxadiazole scaffold that dysregulates ESX-5 protein secretion.

## 1. Introduction

*Mycobacterium tuberculosis* (Mtb), the causative agent of tuberculosis, is one of the deadliest infectious disease, with fatalities significantly increasing due to the impact of the COVID-19 pandemic in recent years [1]. Mycobacteria possess a unique cell envelope structure different from Gram-positive and Gram-negative bacteria [2]. To transport proteins across this complex envelope, mycobacteria employ type VII secretion systems [3,4]. In total, Mtb uses five separate type VII secretion systems, named ESX-1 through ESX-5, which are required for specific functions [5]. Two of these type VII secretion systems, ESX-3 and ESX-5, are essential for bacterial growth in vitro and therefore are interesting targets for developing new antibiotics [6]. The essentiality of ESX-5 can be overcome by the heterologous expression of *mspA*, which encodes a porin derived from fast-growing *M. smegmatis* [7]. In line with this observation, ESX-5 substrates have been identified as nutrient transporters [7]. Other roles of ESX-5 substrates include bacterial capsule integrity, virulence, and immunomodulation [8,9]. The ESX-5 system mediates the secretion of many proteins of the PE/PPE protein families, which contain Pro-Glu (PE domain) or Pro-Pro-Glu (PPE domain) at the N-terminus [10]. PE_PGRS is a notable subclass of PE proteins, which consists of a PE domain followed by a polymorphic GC-rich sequence domain coding for glycine-rich repeats [11]. One of the best-studied ESX-5 substrates is the lipase LipY (Rv3097c). LipY possesses a lipase domain at the C-terminus and has been shown to hydrolyze long-chain triacylglycerol [12,13]. This lipase has been proposed to play a role during latency, as the *lipY*-disrupted Mtb mutant failed to escape the dormancy stage in an in vitro granuloma model [14]. It has been shown that Mtb LipY could be secreted onto the bacterial surface in an ESX-5-dependent manner in both Mtb and *Mycobacterium marinum* [12]. Upon transport to the surface, the PE domain is proteolytically cleaved from LipY by the surface protease PecA [15].

*M. marinum* is a species within the group of nontuberculous mycobacteria and a close genetic relative of Mtb with more than 85% nucleotide identity [16]. *M. marinum* and Mtb are slow-growing mycobacteria; however, with a generation time of six to eight hours, *M. marinum* grows significantly faster than Mtb (20 h generation time) [17]. The infection of zebrafish (*Danio rerio*) with *M. marinum* elicits a disease that shares many features with human tuberculosis caused by Mtb [18]. *M. marinum* has been exploited to investigate tuberculosis pathogenesis, mycobacterial virulence, and high-throughput screening of potential anti-tubercular compounds [18,19,20,21,22,23]. However, the susceptibility profile of *M. marinum* towards anti-tubercular prodrugs, such as isoniazid or ethionamide, is highly different compared to Mtb [24]. In addition, *M. marinum* cannot infect mammalian hosts efficiently like Mtb due to its lower optimal growth temperature of 30 °C. Like Mtb, the genome of *M. marinum* encodes for the ESX-5 secretion system [9]. Previously, we exploited LipY’s ESX-5-dependent secretion and its enzymatic activity to establish a high-throughput screening assay for potential ESX-5 secretion inhibitors in *M. marinum* [25]. In this screening, we identified a 1,2,4-oxadiazole molecule that showed significantly reduced LipY reporter activity. In the present study, we further investigated the effect of this scaffold on ESX-5 secretion activity and pathogenicity. Moreover, the impact of several structural modifications was studied to explore the structure–activity relationship.

## 2. Materials and Methods

### 2.1. Bacterial Strains, Cell Lines, and Culture Conditions

Bacterial strains and plasmids used in this study are listed in Tables S1 and S2. *M. marinum* M^USA^ and Mtb H37Rv were routinely cultured in Middlebrook 7H9 medium (Difco, BD, NJ, USA), supplemented with 0.2% glycerol, 10% ADS (0.5% bovine serum albumin, 0.2% dextrose, and 0.085% sodium chloride), 0.02% tyloxapol with the addition of kanamycin (25 μg/mL) or hygromycin (50 μg/mL) when appropriate. *M. marinum* was grown at 30 °C while Mtb was grown at 37 °C. THP-1 cells (ATCC, TIB-202) were cultured in RPMI medium with GlutaMAX (Gibco, Thermo Fisher Scientific, MA, USA) supplemented with 10% fetal bovine serum (FBS) at 37 °C with 5% CO_2_.

### 2.2. Reagents and Chemical Synthesis

Isoniazid (INH), ethionamide (ETH), and paraoxon (POX) were purchased from Sigma-Aldrich (MO, USA). The synthesis and characterization of the described 1,2,4-oxadiazoles can be found in the Supplementary Information. All antibiotics and test compounds were solubilized in DMSO prior to any described biological assays below.

### 2.3. Bacterial Susceptibility Assay and Cytotoxicity Testing

Test compounds were 2-fold serial diluted in 96-well plate, flat bottom (Corning, New York, NY, USA). *M. marinum* or Mtb were grown to mid-log phase, washed, resuspended in growth medium, and added to the 96-well plates at the final OD_600_ of 0.001. Plates were sealed and incubated for 4 days at 30 °C for *M. marinum* or at 37 °C for Mtb for 7 days. Finally, 20 µL of resazurin solution ([0.025% (*w*/*v*) resazurin sodium salt and 20% Tween 80 in the ratio of 3:1)] were added to each well. After color conversion, fluorescent intensity was measured by plate reader Synergy H1 (Biotek, VT, USA) (excitation 560 nm, emission 590 nm).

For cytotoxicity testing, compound **36** derivatives were diluted in RPMI-Max medium with 10% FBS, resuspended in 96-well plates, and incubated with THP-1 cells for 3 days at 37 °C. After incubation, the development solution (0.025% resazurin sodium salt (*w*/*v*) and 20% Tween 80 in the ratio 3:1] was added to each well for 4 h at 37 °C. The fluorescent intensity was measured using a plate reader (Synergy H1, Biotek), (excitation 560 nm, emission 590 nm).

### 2.4. Lipase Activity Assay

*M. marinum* transformed with pSMT3-lipY-mspA was cultured in 7H9 medium supplemented with 10% ADS and 0.05% Tween-80. Test compounds were serially diluted in the same medium in 96-well plates, flat bottom (Corning). Subsequently, bacteria were washed, suspended, and added to the plates at the final OD_600_ of 0.001 at a total volume of 160 µL. Plates were sealed and incubated for 4 days at 30 °C. Finally, 20 µL of development solution [10 mg/mL BSA, 120 mM NaCl, 50 mM Tris, pH 7.5, 1% Triton X-100, 4 μM DGGR (1,2-di-O-lauryl-*rac*-glycero-3-(glutaric acid 6-methylresorufin ester)) were added and incubated for 3 h. Fluorescent intensity was measured using the plate reader Synergy H1 (Biotek, VT, USA) (excitation 530 nm, emission 600 nm). If needed, bacterial susceptibility assays were performed by the addition of resazurin solution to the wells, as described above.

### 2.5. Protein Secretion Analysis

*M. marinum* M^USA^ wt strain or wt transformed with pSMT3-lipY-mspA were inoculated in 7H9 medium (10% ADS, 0.05% Tween-80) at the OD_600_ of 0.05. The cultures were allowed to grow for 72 h before the cells were washed and reinoculated at an OD_600_ of 0.05 in 7H9, supplemented with compound **36** derivatives at the indicated concentration or DMSO for 72 h. The bacteria were harvested, washed with PBS with Tween, and grown for additional 24 h in 7H9 medium without ADS, supplemented with 0.05% Tween-80. Subsequently, bacterial cells and culture supernatant were separated for further analysis. The bacterial cells were disrupted by bead-beating using 100 µm silica beads (BioSpec, Oklahoma, USA). Proteins in the culture supernatant were precipitated by the addition of trichloroacetic acid (TCA) to 10% (*w*/*v*) (Sigma-Aldrich, Missouri, USA) and the addition of tRNA 20 mg/mL (Roche, Basel, Switzerland). After allowing the proteins to precipitate for 45 min on ice, the proteins were harvested by centrifugation (21,000× *g*, 15 min at 4 °C), and the resulting pellet was washed with cold acetone. Proteins in each fraction were separated by SDS-PAGE (12.5% gel) and transferred to nitrocellulose membranes. The membranes were blocked with 5% skimmed milk in PBS, and immunostaining of protein with different antibodies was carried out (anti-PE_PGRS; 7C4.1F7, anti-GroEL2; Cs44, anti-EsxA; Hyb 76-8 and anti-HA.11; Covance). Membranes were washed with TBST and incubated with secondary antibodies, goat anti-mouse (American Qualex, CA, USA), or goat anti-rabbit (Rockland, Pennsylvania, USA) conjugated with horseradish peroxidase. Finally, proteins were visualized with chemiluminescence using Lumi-Light Western Blotting Substrate (Roche).

### 2.6. Mtb Macrophage Infection Experiments

Infection of THP-1 cells with Mtb harboring vector pTetDuo and compound treatment was performed as previously described [23]. Briefly, THP-1 cells were seeded in 96-well plates (Corning, New York, NY, USA) at 10^5^ cells/well and differentiated into macrophage-like cells by adding and incubating with phorbol-12-myristate-13-acetate (PMA) (25 ng/mL) (Sigma-Aldrich, St. Louis, Missouri, USA) at 37 °C. After 48 h, cells were infected with Mtb harboring pTetDuo at the multiplicity of infection (MOI) of 5. After 3 h of incubation, extracellular bacteria were treated with gentamycin (25 µg/mL) for 1 h. Subsequently, the medium was replenished with a medium having the diluted compounds at indicated concentrations. Plates were incubated for 4 days before anhydrotetracycline (ATc) (100 ng/mL) (IBA Lifesciences, Gottingen, Germany) was added, and plates were incubated for additional 24 h. The medium was then replaced with paraformaldehyde diluted in PBS [3.2%(*w*/*v*)] for fixation. Finally, quenching/staining solution [0.1 M glycine, 0.2% (*w*/*v*) Triton X-100 and Hoechst dye 1:500 in PBS] was added to the plates and incubated for 1 hour. Plates were then imaged by Olympus IX83 fluorescence microscope (Olympus, Tokyo, Japan) and infection efficiency was quantified using a custom pipeline of Cellprofiller 3.19 (Broad Institute, Cambridge, MA, USA).

### 2.7. Proteomic Analysis

*M. marinum* culture supernatant treated with DMSO or compound **36.3** were separated by SDS-PAGE (10%). The gels were stained using Coomassie Brilliant Blue Staining Solution G-250 (Thermo Fisher Scientific, Massachusetts, USA). The areas on the gel corresponding to more and less than 130 kDa were independently excised, washed, and further processed for in-gel digestion. Peptides were eluted from gel samples and further processed and analyzed by Ultimate 3000 nanoLC-MS/MS system (Dionex LC531 Packings, Thermo Fisher Scientific, Massachusetts, USA), as previously described [7].

### 2.8. Construction of the Reporter Plasmid and Reporter Assay

The promoter sequence of *mmar_5294* (500 bp upstream of the start codon) was amplified using primers FW-pro-mmar5294 and RV-pro-mmar5294 and genomic DNA of *M. marinum* M^USA^. The gene *tdTomato* was amplified using primers FW-tdTomato and RV-tdTomato and plasmid pML2424 as a DNA template (Table S3). Amplified products of *mmar_5294* promoter sequence and *tdTomato* were ligated into pMN016 plasmid digested with XbaI and HindIII using Infusion HD Cloning kit (Takara Bio, Shiga, Japan), resulting in plasmid pMN016-pro*_mmar_5294_*-tdTomato. Amplified regions were sequenced and transformed into *M. marinum* M^USA^ strains.

Bacterial cells of the *M. marinum* reporter strain harboring plasmid pMN016-pro*_mmar_5294_*-tdTomato were grown to the mid-logarithmic phase and collected by centrifugation. Subsequently, bacterial cells were diluted in 7H9 medium (10% ADS, 0.02% tyloxapol) to a final OD_600_ of 0.001 in 96-well plates containing 2-fold dilutions of compound **36** derivatives or ethionamide (ETH). Plates were sealed and incubated at 30 °C. The fluorescent intensity was measured daily for 3 days using plate reader Synergy H1 (Biotek) (excitation 554 nm, emission 581 nm).

### 2.9. Zebrafish Embryos Infection Experiments and Data Analysis

Zebrafish handling was conducted in compliance with local animal welfare laws [Animal Experimental Licensing Committee, Dier Experimenten Commissie (DEC)]. All zebrafish protocols adhere to the international guidelines from the EU Animal Protection Directive 86/609/EEC. Injection stock of *M. marinum* M^USA^ strain (transformed with pMS2-*tdTomato*) was prepared in PBS with 20% glycerol and stored at −80 °C until further use. Transparent casper zebrafish (*Danio rerio*) embryos were yolk-infected with 1 nL suspension of *M.marinum*-tdTomato (150 CFU) and green fluorescent dye fluorescein (2.5 µg/mL) one-hour post-fertilization [26]. The injection was performed using an automated microinjection system (Life Science Methods BV, Leiden, The Netherlands), as described previously [23]. Successfully injected embryos were selected and incubated for 24 h in E3 medium (5.0 mM NaCl, 0.17 mM KCl, 0.33 mM CaCl*2H_2_O, 0.33 mM MgCl_2_*6H_2_O) supplemented with 0.3 mg/L methylene blue at 31 °C. Next, embryos were transferred as 12 embryos per well in a 12-well plate and treated by immersion of infected embryos in chorion water (60 µg/mL instant ocean sea salts), supplemented with test compounds. After 3 days of treatment at 28 °C, the embryos were anesthetized in 0.02% (*w*/*v*) buffered 3-aminobenzoic acid methyl ester (pH 7.0) (Tricaine; Sigma-Aldrich), and imaged using Olympus IX83 fluorescence microscope (4× objective magnification, Hamamatsu ORCA-Flash 4.0 camera, excitation 550 nm/emission at 610 nm) (Olympus, Tokyo, Japan). The images were analyzed with CellProfiler 3.19 (Broad Institute, Cambridge, MA, USA) using a pipeline that quantifies the intensity of pixels per embryo, which was used as a readout for a total bacterial burden per embryo. The signal from the non-infected group was used to set the detection threshold, and all data points equal to 0 were set to 1 to allow for log-10 transformation of the data points. The statistical analysis was performed using GraphPad Prism version 8.1.1 (GraphPad Software Inc., San Diego, CA, USA) on log-transformed values. A one-way analysis of variance (ANOVA) and Dunnett’s post hoc test was used to compare the effect of compound treatment with the DMSO-treated control sample.

## 3. Results

### 3.1. Characterization of 1,2,4-oxadiazole Derivatives Using ESX-5 Secretion Reporter Assay

**Figure 1:**
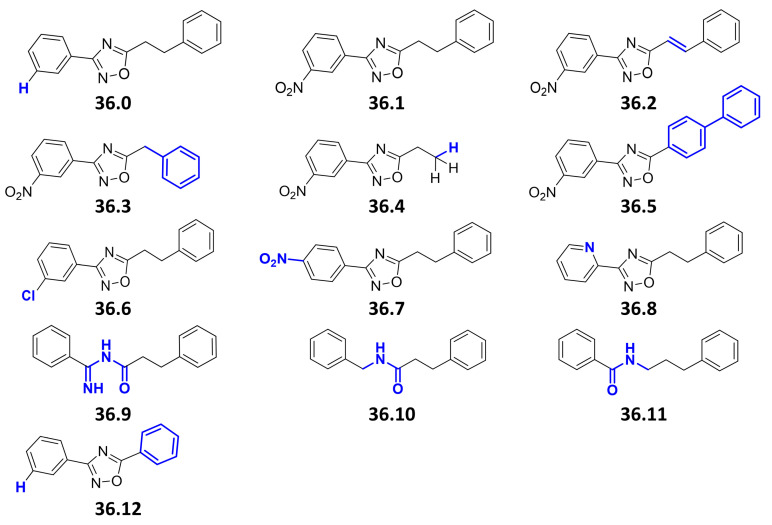
**Chemical structures of compound 36 derivatives**. Chemical variations between the original hit compound 36.1 and its newly synthesized derivatives are highlighted in blue. The compounds **36.0; 36.1; 36.2; 36.3; 36.4; 36.5; 36.6; 36.7; 36.8; 36.9** showed a MIC_50_ > 40 µM when tested against Mtb H37Rv or *M. marinum* M^USA^. No toxicity to THP-1 cells was observed at concentrations up to 40 µM. Compounds **36.10, 36.11,** and **36.12** were not tested.

We previously performed a compound screen to identify ESX-5 secretion inhibitors using *M. marinum* overexpressing the ESX-5 substrate LipY as a reporter for ESX-5 activity [25]. This strain was modified to produce the porin MspA to overcome the essentiality of ESX-5 of Mycobacteria [7]. Following the screening, the 3-(3-nitrophenyl)-1,2,4-oxadiazole **36.1** was selected as a promising hit (Figure 1). We then identified a facile synthesis route to **36.1** from 3-nitrobenzamidoxime and hydrocinnamoyl chloride (59% over three steps) and confirmed its identity spectroscopically (Figure S1). The newly synthesized compound displayed identical activity in the LipY reporter assay as the original compound **36.1** from the library. Subsequently, 13 derivatives were synthesized, to vary four structural features of the parent compound (i.e., the nitro, 3-aryl, oxadiazole, and ethylene groups) (Figure 1). We first removed the nitro group (**36.0**), as it can generate highly reactive intermediates in vivo with high activity but also toxicity and mutagenicity [27]. Additionally, to investigate the importance of the electron-withdrawing nature of the 3-nitro substituent, groups with a similar effect were introduced in the 2-,3- and 4-positions (**36.6**, **36.7**, **36.8**). The phenethyl group of **36.1** was replaced by styryl (**36.2**) or truncated to a benzyl, a free ethyl, and a phenyl group (**36.3**, **36.4**, **36.12**). The 1,2,4-oxadiazole was replaced by bioisosteric alternatives (**36.9**, **36.10**, **36.11**) [28].

**Figure 2:**
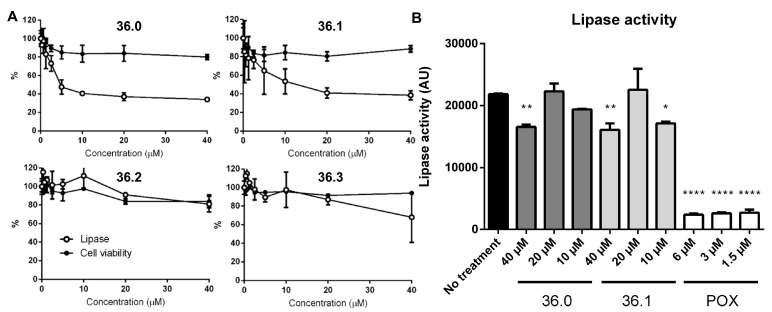
**Compound 36 derivatives reduce LipY activity.** (**A**) *M. marinum* overexpressing *lipY* and *mspA* was grown in the presence of test compounds. After 3 days, lipase activity was measured using DGGR as a substrate. Subsequently, cell viability was determined by REMA. (**B**) *M. marinum* overexpressing *lipY* and *mspA* was incubated with compounds **36.0, 36.1,** or lipase inhibitor paraoxon (POX) for 2 h. Afterward, lipase activity was measured using the lipase assay (DGGR substrate). One-way ANOVA calculated statistical significance with Dunnett post-hoc test by comparing lipase activity from the untreated group with each treatment group (* *p*≤ 0.05, ** *p* ≤ 0.01, **** *p* ≤ 0.0001).

*M. marinum* bacteria were grown in the presence of the new compound derivatives for several days before the overall amount of secreted reporter lipase was indirectly quantified by adding a fluorescent lipase substrate. As expected, we observed lower lipase activity in the presence of the original 1,2,4-oxadiazole (**36.1**) in a dose-dependent manner, suggesting reduced amounts of active LipY on the bacterial cell surface (Figure 2A). Among the other derivatives, lower lipase activity was found for bacteria grown in the presence of **36.0**, which notably lacked the *meta*-nitro substituent with respect to **36.1**. This effect was lost by additional changes to the oxadiazole core (**36.9–36.11**). We also observed slightly reduced lipase activity for the *para*-nitrophenyl substituted **36.7** and the pyridyl derivative **36.8**, while other derivatives showed no significant effect on lipase activity (Figure S2). To verify whether compounds **36.0** and **36.1** directly inhibited the lipase activity, we exposed the *M. marinum* LipY reporter strain only for a short time to the compounds instead of growing the bacteria in the presence of the compounds for several days. Using this approach, sufficient LipY enzyme was located on the bacterial surface, and activity could only be blocked if compounds directly blocked the enzyme. As a positive control, we used the lipase inhibitor paraoxon (POX) [15]. While less than 10% activity was detected for POX at 1.5 µM, only a minor reduction of lipase activity with no dose-response relationship was observed for compounds **36.0** and **36.1** at 40 µM (Figure 2B). These data suggest that the original 1,2,4-oxadiazole **36.1** and its derivative **36.0** both affect ESX-5-dependent secretion and not the LipY lipase reporter. None of the tested compounds affected bacterial viability at the tested concentrations.

### 3.2. The 1,2,4-oxadiazole Derivatives 36.1 and 36.3 Elicit Hypersecretion of ESX-5 PE_PGRS Substrates

**Figure 3:**
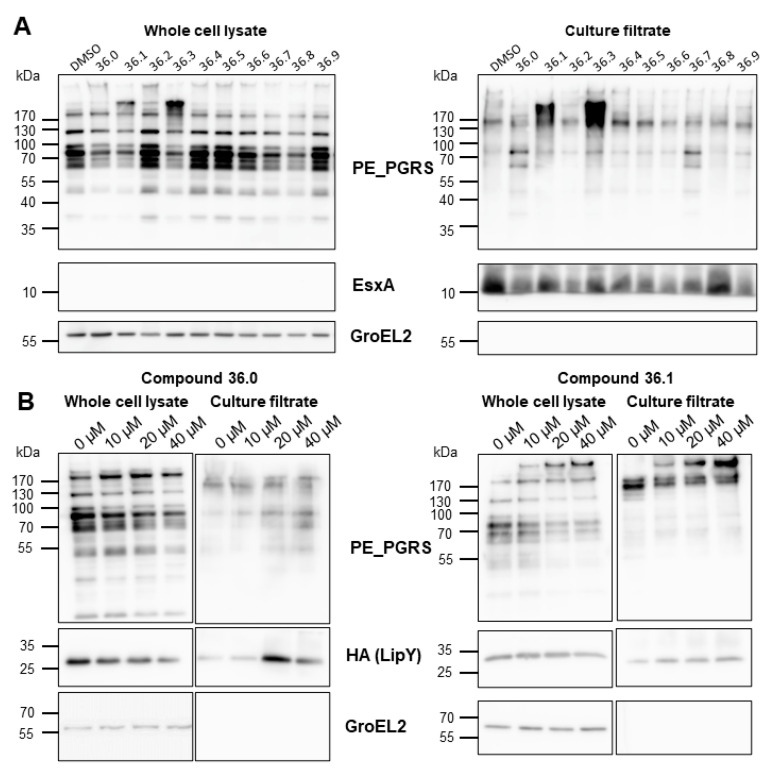
**Compound 36 derivatives induced hypersecretion of ESX-5 substrates.** (**A**) Secretion analysis of *M. marinum* transformed with pSMT3-lipY-mspA treated with indicated compounds at 20 µM. Whole-cell lysates and culture filtrate were separated by SDS-PAGE and analyzed with antibodies against PE_PGRS, EsxA, or GroEL2. (**B**) Secretion analysis *M. marinum* overexpressing *lipY* and *mspA* treated with compounds **36.0** and **36.1** in dose-response from 0 µM to 40 µM. Whole-cell lysates and culture filtrate were separated and analyzed with antibodies against PE_PGRS, HA tag, or GroEL2.

We showed that compounds **36.0** and **36.1** did not directly inhibit the lipase activity of LipY and hypothesized that the observed reduced lipase activity might have resulted from reduced ESX-5 activity, resulting in a lower amount of surface localized LipY. However, we did not see a reduction in the amount of secreted LipY into the culture supernatant. Instead, LipY was present in slightly higher levels in the supernatant with increasing concentrations of **36.0** and **36.1**. The molecular weight of the detected LipY protein correlated with the processed form of the protein, in which the PE domain was cleaved after its transport to the cell envelope. We then investigated whether any of the 1,2,4-oxadiazole derivatives affected the secretion of other ESX-5 substrates, focusing on PE_PGRS, a major group of ESX-5 substrates. To our surprise, we did not see a decreased amount of these ESX-5 substrates but instead observed increased PE_PGRS protein secretion in the supernatant fraction for several compounds. The original compound **36.1** and its truncated derivative **36.3** mainly induced hypersecretion of the PE_PGRS with an apparent molecular weight of 130 kDa (Figure 3A). Conversely, compound **36.0**, which lacked the nitro group and had the most substantial effect on lipase activity, hardly impacted PE_PGRS secretion; only distinct PE_PGRS proteins with an apparent molecular weight of approximately 70 kDa were slightly increased. A dose-response secretion analysis further verified the contrasting PE_PGRS patterns elicited by **36.0** and **36.1** up to 40 µM. As expected, the hypersecretion of PE_PGRS in the **36.1**-treated *M. marinum* was more pronounced with increasing concentration (Figure 3B). To investigate specificity, we determined the effect of those compounds on ESX-1 secretion by examining the ESX-1 substrate EsxA. In contrast to the ESX-5 substrates, only a modest effect on EsxA secretion was detected. The control protein GroEL2 was not detected in the culture filtrate fraction, indicating that the compounds did not lyse the bacterial cells. These experiments showed two crucial effects. Firstly, the 1,2,4-oxadiazoles did not inhibit the ESX-5 secretion process itself. Secondly, they had a dual effect on ESX-5 substrates, i.e., they reduced the amount of active LipY and increased the secretion of specific PE_PGRS proteins. Both effects were seen for the parent compound **36.1**, but these effects could be achieved independently in several effective derivatives, e.g., **36.0**, which only blocked LipY activity, and **36.3**, which only induced PE_PGRS secretion. These effects generally correlated to two structural features in the tested compounds: the 5-phenethyl substituent (**36.0**, **36.1, 36.7, 36.8**) for LipY inhibition, and the 3-nitrophenyl group (**36.1**, **36.3**) for enhancement of PE_PGRS secretion.

### 3.3. The 1,2,4-oxadiazole Derivatives 36.1 and 36.3 Induced the Overexpression of PE_PGRS MMAR_5294

To further investigate the mechanism of action of our 1,2,4-oxadiazoles that affect ESX-5 activity, we sought to explore the identity of the PE_PGRS protein(s) that were secreted in higher amounts in bacteria treated with compound **36.3**. We isolated the culture supernatant fraction from *M. marinum* treated with **36.3**, separated the proteins by SDS-PAGE, and subjected proteins above 130 kDa to proteomic analysis. We identified seven proteins that were present in increased amounts. Surprisingly, non-ribosomal peptide synthetases showed the most considerable increase (Table 1). Among the seven proteins, one PE_PGRS protein, MMAR_5294, was present in the supernatant derived from samples treated with compound **36.3** and not in the control sample. The predicted molecular weight of this protein was 183 kDa, which matched the apparent molecular weight of the induced protein observed on Western blot analysis.

**Figure 4:**
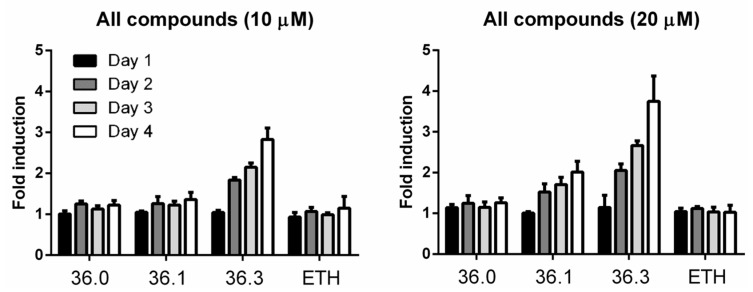
**Compounds 36.1 and 36.3 induce the expression of *mmar_5294*.***M. marinum* strain harboring a transcriptional reporter construct for gene *mmar_5294* was treated with different amounts of compounds **36.0**, **36.1**, **36.3**, or ETH. Each day, fluorescent intensity (excitation 554 nm/emission 581) was measured. Fold induction was calculated by dividing fluorescent intensity from indicated treated samples to untreated samples.

The hypersecretion of MMAR_5294 induced by **36.3** was possibly due to induced expression levels. We investigated this by generating a *M. marinum* reporter strain setting the sequence of *tdTomato* (RFP) under the control of the *mmar_5294* promoter. The reporter strain was grown in the presence of compounds **36.0**, **36.1,** and **36.3**, and the fluorescent signal of Tdtomato was followed for four days. The antibiotic ethionamide (ETH) was included as a negative control. We observed increasing fluorescent intensity when treated with **36.3** following each day of incubation, with the highest increase of approximately 4-fold compared to untreated samples on the final day at 20 µM. The increased reporter signal was also dose-dependent with compound **36.1**, although to a lower extent (Figure 4). Importantly, for **36.0** and ETH, no significant change in reporter activity was detected throughout three days, indicating that this effect was specific for the compounds that showed increased PE_PGRS secretion (Figure 4). In conclusion, compounds **36.3** and **36.1**, but not **36.0**, induced the expression of PE_PGRS protein MMAR_5294 in a dose-dependent manner.

### 3.4. 1,2,4-oxadiazoles Display Significant in vivo Activity

Although our investigated compounds were not direct ESX-5 secretion inhibitors but did affect the accumulation of active ESX-5 substrates on the cell surface, we still were interested in testing their effect on mycobacterial growth. We tested the impact of our compounds on the growth of our model strain, *M. marinum*, and on Mtb H37Rv, using a resazurin microtiter assay. None of the compounds could reduce the bacterial viability of *M. marinum* or Mtb by more than 50% at the highest tested concentration (40 µM) (Figure 1). We next tested our two most active compounds with a different selective effect, i.e., the lipase affecting **36.0** and the PE_PGRS inducing **36.3,** in Mtb-infected macrophages. We observed that compound **36.3** showed a modest decrease in bacterial infection at 30 µM, while the reduction of bacterial infection by compound **36.0** was insignificant (Figure S3). Using a resazurin reduction assay, we determined that none of the compounds showed toxicity against THP-1 cells up to 40 µM (Figure 1).

**Figure 5:**
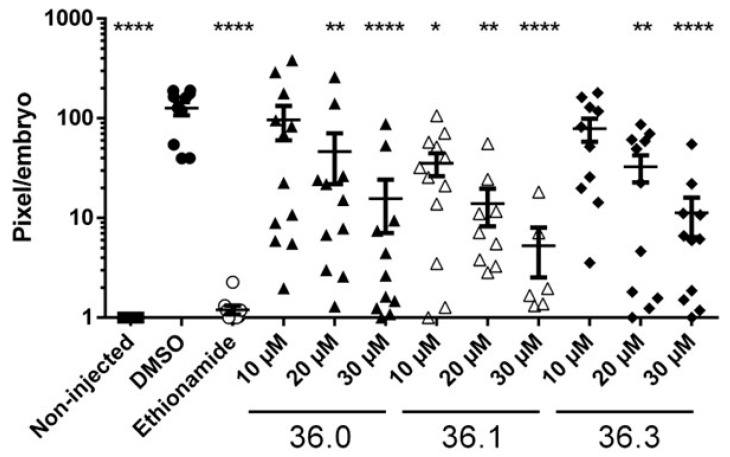
**Three 1,2,4-oxadiazole derivatives reduced bacterial outgrowth in infected zebrafish.** Zebrafish were infected via yolk injection with Tdtomato-labeled *M. marinum* and treated with tested compounds at indicated concentrations with 12 embryos per group. At 3 days post-treatment, infection efficiency was monitored by determining the fluorescent intensity of Tdtomato. Deformed or dead fish were removed from the analysis. Viable embryos were scored, with each dot representing a single viable embryo. Statistical significance was determined by one-way ANOVA with Dunnett post hoc test by comparing the signal from the DMSO-treated control sample with non-injected or each treatment group (* *p *≤ 0.05, ** *p *≤ 0.01, **** *p *≤ 0.0001).

Finally, the three 1,2,4-oxadiazole derivatives with significant activities, **36.0**, **36.1**, and **36.3**, were tested for in vivo activity in our zebrafish (*Danio rerio*) infection model [23]. The zebrafish were injected with *M. marinum* expressing fluorescent Tdtomato using an automated robotic microinjector at 2 h post-fertilization. After 24 h, the investigated compounds were added to the chorion water and the bacterial burden was monitored at 72-h post-treatment. Interestingly, we observed that the bacterial burden in zebrafish was significantly decreased upon treatment with **36.0**, **36.1**, and **36.3**. This could mean that both phenotypes, i.e., reduced LipY activity and PE_PGRS induction, resulted in significantly reduced outgrowth in vivo of at least a factor 10. The parent compound **36.1**, which induced both phenotypes, was most effective with a significant reduction of bacterial outgrowth at 10 µM. Moderate toxicity was observed at 30 µM with only approximately half of the embryos surviving the treatment during infection (Figure 5). All other 1,2,4-oxadiazoles of this study were also tested in the zebrafish model at 10 µM or 30 µM, but no significant reduction of mycobacterial infection was detected for these compounds (Figure S4). In conclusion, the three 1,2,4-oxadiazoles, which possessed activities against the ESX-5 secretion system, were also the only compounds with in vivo activity in our study.

## 4. Discussion

Type VII secretion systems are important for pathogenic mycobacteria. The ESX-1 system is required for escape from the phagosome in host cells [29,30]. However, ESX-1 is not essential for mycobacterial viability. In contrast, ESX-3 and ESX-5 are both required for mycobacterial survival in vitro and could therefore be viable targets for chemical interference [31].

In this study, we initially confirmed the activity of a potential ESX-5 effector 3-(3-nitrophenyl)-5-(phenethyl)-1,2,4-oxadiazole (**36.1**). This compound blocked the presence of active LipY lipase at the bacterium’s surface but did not directly block the lipase itself. Hence it was assumed that it could block the ESX-5 secretion process. As 1,2,4-oxadiazole compounds have been reported to possess many biological activities, including antimycobacterial activity [32,33,34,35], we decided to characterize the effect of this compound further. In addition, 13 derivatives of the original 1,2,4-oxadiazole were synthesized and tested to study the structure-activity relationship of **36.1** by varying structural features of the parent compound. The nitro and phenethyl groups were found to be responsible for two observed effects on ESX-5 secretion by respectively reducing the presence of active LipY on the bacterial surface or enhancing PE_PGRS secretion. The effect of lowered LipY activity did not correlate with reduced secretion; only the parent compound, with both the nitro- and phenethyl substituents, showed both activities. The effect of **36.0** and **36.1** on LipY might correlate with their phenethyl groups, as even minor changes to this group resulted in the loss of the observed phenotype, as strikingly demonstrated by **36.2** and **36.3**. Nevertheless, no effects were observed for the phenethyl-derivatives **36.6**-**36.8**, which may indicate that the specific changes in their aryl groups were too substantial, or such aryl variations were poorly tolerated in general. In addition, the overall phenotype of LipY secretion and activity induced by the compounds could be because the secreted LipY proteins were not correctly folded and consequently inactive. Future structural studies should be performed to clarify this possibility. Moreover, the observed effects on LipY and hypersecretion of the ESX-5 substrates PE_PGRS were not due to inhibition of substrate processing since no molecular weight shifts of PE_PGRS proteins or LipY were detected, as seen in a previous study [15].

Next, we identified a strongly secreted high-molecular-weight PE_PGRS protein, MMAR_5294, in the secreted fraction of *M. marinum* induced by **36.3**. This protein was shown to be up-regulated by **36.3** and **36.1***,* but not **36.0,** in a reporter assay, which agreed with the PE_PGRS hypersecretion pattern in the secretion analysis. More importantly, the results of the reporter assay in combination with proteomic analysis suggested that up-regulation of MMAR_5294 occurred in particular stress conditions triggered by **36.1** and **36.3**. Previously, the expression of several other high-molecular-weight proteins (PE_PGRS27 and PE_PGRS50) has not been detected in general growth culture, showing that mycobacterial species do not favor expressing high amounts of these proteins under standard culture conditions [36]. Besides, MMAR_5294 has not been listed as a putative virulence gene required for *M. marinum* survival in macrophages [37]. MMAR_5294 consists in a total of 2433 amino acids and is homologous to PE_PGRS57 (Rv3514) in Mtb. This PE_PGRS protein has more amino acids and a higher molecular weight than other PE_PGRS proteins [38]. Thus far, no studies have been performed to determine the function of these large PE_PGRS proteins.

Interestingly, a recent study identified mutations within PE_PGRS57 in Mtb that are associated with resistance to a novel anti-tubercular compound [39]. However, since this protein is not essential for Mtb in vitro, it could be that this compound has an additional target [40,41]. In other studies, PE_PGRS57 was only mentioned as harboring mutations in several clinical avirulent Mtb H37Rv strains, suggesting it might be related to infection efficiency in the clinical setting [42]. Overall, the activity of **36.1** and **36.3**, but not **36.0**, emphasized the contribution of the *meta* NO_2_ group in promoting this distinct phenotype. No effects were observed for derivatives with the nitro group at a different position (**36.7**) or similar electronic properties (**36.6–36.8**). Drastic changes to the rest of the compound were not allowed (**36.4**, **36.5**, **36.12**). Although all tested compound **36** derivatives did not significantly affect the bacterial growth of *M. marinum* and Mtb under culture conditions, we observed a significant reduction in bacterial burden in *M. marinum*-infected zebrafish models during treatment with the three derivatives **36.0**, **36.1**, and **36.3**. These data also indicate that the three compounds do not target the essential secretion system ESX-5 but are more likely to target several ESX-5-dependent substrates. Since the observed bacterial phenotypes in the presence of the three most active compounds differed, it is tempting to speculate that the reduction of mycobacterial burden during infection could be attributed to different mechanisms for each effect. Compound **36.0** was demonstrated to reduce LipY activity. LipY was proven to be a virulence factor in Mtb, and Mtb overexpressing LipY triggered a higher mortality rate in the murine model [43]. These studies and our data suggest that **36.0** suppresses *M. marinum* virulence in vivo by inhibiting its LipY lipase activity. On the other hand, **36.3** induced specific PE_PGRS overexpression and hypersecretion with a much smaller effect on LipY. Overexpression of several PE_PGRS could lead to cell wall remodeling, resulting in envelope stress and eventual virulence reduction in vivo [44,45]. Indeed, PE_PGRS proteins like PE_PGRS26 are found to be significantly down-regulated during in vivo infection [36]. Therefore, we postulate that PE_PGRS overexpression triggered by **36.3**, including MMAR_5294, elicits envelope stress in *M. marinum,* causing virulence attenuation in the zebrafish model. In addition, overexpression of MMAR_5294 might cause saturation of protein export mediated by the ESX-5 secretion system, which in turn influences the transport of other important ESX-5 substrates. This phenomenon has been reported for the substrates of the Tat secretion system in Mtb and *E. coli* [46,47]. However, more studies are needed to clarify the consequences of high-molecular-weight PE_PGRS overexpression on mycobacterial cell envelope integrity or saturation of the secretion pathway. Because compound **36.1** demonstrated both properties, including suppression of LipY activity and PE_PGRS overexpression, the attenuation of *M. marinum* in the infected zebrafish model treated with **36.1** can be attributed to both mechanisms. Other 1,2,4-oxadiazole derivatives with no or mild impact on either LipY activity or PE_PGRS secretion showed no in vivo activity in the zebrafish model. Taken together, we proposed that our three studied 1,2,4-oxadiazoles (**36.0**, **36.1**, **36.3**) can dysregulate the normal secretion patterns of the ESX-5 system in different ways, which eventually lead to reduced outgrowth in vivo.

In conclusion, we identified novel 1,2,4-oxadiazole-containing compounds that can dysregulate ESX-5 secretion and induce overexpression and secretion of a high-molecular-weight PE_PGRS and/or render the ESX-5 substrate LipY on the surface unfunctional. Our results also imply that the balanced and tight regulation of ESX-5 secretion system activity is required to maintain mycobacterial survival in the host.

## Figures and Tables

**Table 1:** Proteins above 130 kDa that were more abundant in the supernatant fraction of compound 36.3-treated *M. marinum* as compared to DMSO treated-*M. marinum* (*p*-value < 0.05 and fold change >1).

Protein	Average Count DMSO-Treated	Average Count 36.3-Treated	Fold Change	*p*-Value
MMAR_3268 MMAR_3271 MMAR_3279 MMAR_0851	0	38.39	∞	0.0004
MMAR_5294	0	13.77	∞	0.0032
CobN	0	7.98	∞	0.0082
MMAR_4621	0	9.605	∞	0.0055
MmpL5	1.11	28.29	25.40	0.001
MMAR_5283	1.11	9.42	8.46	0.0322
DnaE1	6.68	33.62	5.03	0.0125

## Data Availability

Data from the proteomic analysis was deposited on the ProteomeXchange Consortium with the dataset identifier PXD037981.

## Supplementary Materials

The following supporting information can be downloaded at: https://www.mdpi.com/article/10.3390/biom13020211/s1, Supplementary Materials and Methods. Figure S1: Optimized synthesis route towards 1,2,4-oxadiazoles 36.0–36.8 and 36.12.; Figure S2: 1,2,4-oxadiazole derivatives show variable inhibition of LipY activity; Figure S3: Compound 36.3 significantly reduces bacterial burden in *M. tuberculosis*-infected macrophages; Figure S4: 1,2,4-oxadiazole derivatives that do not show activity in the zebrafish infection model; Table S1: Bacterial strains used in the study; Table S2: Plasmids used in the study; Table S3: Primers used in the study [48,49,50,51,52,53,54,55,56].
