# Supplementary material for: Dysregulation of Mycobacterium marinum ESX-5 Secretion by Novel 1,2,4-oxadiazoles
Source: Biomolecules. 2023 Jan 21;13(2):211. doi: 10.3390/biom13020211 (PMC9953084; doi:10.3390/biom13020211)
Supplement: Supplementary file 1 [file biomolecules-13-00211-s001.zip › biomolecules-2146477-supplementary.pdf]

Supplementary Information for

"Dysregulation of *Mycobacterium marinum* ESX-5 Secretion by Novel 1,2,4-oxadiazoles"

Vien Q. T. Ho[1], Mark K. Rong[2], Eva Habjan[1], Samantha D. Bommer[2], Thang V. Pham[3], Sander R. Piersma[3], Wilbert Bitter[1,4], Eelco Ruijter[2], Alexander Speer[1]*

[1] Department of Medical Microbiology and Infection Control, Amsterdam UMC, Vrije Universiteit Medical Center, 1081HV, Amsterdam, The Netherlands

[2] Department of Chemistry and Pharmaceutical Sciences, Amsterdam Institute of Molecular and Life Sciences (AIMMS), Vrije Universiteit Amsterdam, 1081HV, Amsterdam, The Netherlands

[3] Department of Medical Oncology, OncoProteomics Laboratory, AmsterdamUMC, Vrije Universiteit Medical Center ,1081HV, Amsterdam, The Netherlands

[4] Department of Molecular Microbiology, Amsterdam Institute of Molecular and Life Sciences (AIMMS), Vrije Universiteit Amsterdam, 1081HV, Amsterdam, The Netherlands

* Correspondence: a.speer@amsterdamumc.nl.

Supplementary materials and methods

General information chemical syntheses

Commercially available reagents and solvents were used as received, unless stated otherwise. Experiments under an atmosphere of dry nitrogen were performed using standard Schlenk-line techniques. Molecular sieves were dried before use (*in vacuo*, 300°C, >18h). Nuclear magnetic resonance (NMR) spectra were recorded on a Bruker Fourier 300 (^{1}H, 300.13 MHz; room temperature), a Bruker Avance II 500 (^{1}H, 500.23 MHz; ^{13}C, 125.78 MHz; room temperature) or a Bruker Avance III 600 instrument (^{1}H, 600.13 MHz; ^{13}C, 150.90 MHz; room temperature). ^{1}H spectra and $^{13}C\{^{1}H\}$ spectra were internally referenced to residual solvent resonances ($CDCl_3$, $\delta(^{1}H)$ 7.26, $\delta(^{13}C\{^{1}H\})$ 77.16; DMSO-d_6 $\delta(^{1}H)$ 2.50). Chemical shifts (δ) are reported in ppm and coupling constants (J)

are quoted in hertz (Hz). High-resolution electrospray ionization (ESI) mass spectrometry was carried out with a Bruker Impact II instrument in positive ion mode (capillary potential of 4500V). Flash chromatography was performed on Silicycle Silica-P Flash Silica Gel (particle size 40-63 µm, pore diameter 60 Å) using the indicated eluent. Thin Layer Chromatography (TLC) was performed using TLC plates from Merck (SiO_2, Kieselgel 60 F254 neutral, on aluminium with fluorescence indicator). Several methods were explored to access **36.0**-**36.8** and **36.12**, of which our general method for **36.1** (**Figure S1**) proved the most convenient to minimize hydrolysis side-reactions and is recommended to use in future studies.

Syntheses Precursors

3-nitrobenzonitrile

Benzonitrile (5.5 mL, 53.3 mmol, 1.0 eq) was dissolved in sulfuric acid (50 mL) and cooled to approximately −25 °C. Nitric acid (10 mL, 224.3 mmol, 4.2 eq) was slowly added dropwise, while the temperature of the reaction mixture was kept below – 8 °C. After 10 minutes, a bright yellow solution was obtained, which was poured onto ice and then isolated by filtration. The resulting light-yellow residue was washed with water and dried overnight to afford 3-nitrobenzonitrile as a light-yellow powder (7.64 g, 51.5 mmol, 97%). ^{1}H NMR (300.13 MHz, $CDCl_3$): δ 8.54 (s, 1H, O_2N-C(C*H*)-C), 8.48 (d, $^3J_{H,H}$= 8.3 Hz, 1H, Ar-*H*), 8.00 (d, $^3J_{H,H}$= 7.7 Hz, 1H, Ar-*H*), 7.74 (t, $^3J_{H,H}$= 8.0 Hz, 1H, O_2N-C-CH-C*H*) (48).

3-nitro-benzamidoxime

3-nitrobenzonitrile (1.48 g, 10.0 mmol, 1.0 eq) was suspended in EtOH (25 mL). Hydroxylamine hydrochloride (1.04 g, 15.0 mmol, 1.5 eq) and NaOH (600 mg, 15.0 mmol, 1.5 eq) were added to the yellow mixture, which was then refluxed for 18h. The yellow suspension was filtered and the bright yellow filtrate was evaporated to give a yellow/orange powder. The powder was then dissolved in THF and filtered. Evaporation of the filtrate provided 3-nitrobenzamidoxime as a dark yellow powder (1.32 g, 7.30 mmol, 73%). ^{1}H NMR (300.13 MHz, DMSO-d_6): δ 10.03 (s, 1H, O*H*), 8.51 (s, 1H, O_2N-C-C(C*H*)-C), 8.24 (d, $^3J_{H,H}$= 8.1 Hz, 1H, Ar-*H*), 8.12 (d, $^3J_{H,H}$= 8.0 Hz, 1H, Ar-*H*), 7.69 (t, $^3J_{H,H}$= 8.0 Hz, 1H, O_2N-C-CH-C*H*), 6.23 (s, 2H, NH_2) (49).

3-chloro-benzamidoxime

3-chlorobenzonitrile (550 mg, 4.0 mmol, 1.0 eq) was dissolved in EtOH (15 mL). NaOH (246 mg, 6.0 mmol, 1.5 eq) and hydroxylamine (417 mg, 6.0 mmol, 1.5 eq) were added and the resulting white suspension was stirred at 120 °C in closed vessel and after 2.5h the suspension was filtered. The filtrate was evaporated to give a mixture of an oil and beige crystals. The oil was dissolved in DCM. After filtration, the DCM was evaporated to give a mixture of white and brown crystals. The white crystals could be dissolved in warm toluene. Evaporation provided 3-chlorobenzamidoxime as a white powder (156 mg, 0.91 mmol, 23%). ^{1}H NMR (300.13 MHz, DMSO-d_6): δ 9.80 (s, 1H, O*H*), 7.70 (s, 1H, Cl-CC(C*H*)-C), 7.64 (d, $^3J_{H,H}$= 7.1 Hz, 1H, Ar-*H*), 7.42-7.37 (m, 2H, Ar-*H*), 5.92 (s, 2H, NH_2) (50).

4-nitro-benzamidoxime

4-nitrobenzonitrile (592 mg, 4.0 mmol, 1.0 eq) was dissolved in EtOH (15 mL) to give a beige solution, which turned orange upon the addition of NaOH (240 mg, 6.0 mmol, 1.5 eq). Next, hydroxylamine hydrochloride (417 mg, 6.0 mmol, 1.5 eq) was added and the mixture turned dark purple. It was refluxed for 2.5h. The resulting orange mixture was evaporated and the resulting solid was washed twice with DCM to provide crude 4-nitrobenzamidoxime as a yellow powder (41 mg, 82 mol% pure (^{1}H NMR), contaminant: NH_4Cl), which was used without further purification (corrected yield: 1.00 mmol, 25%). ^{1}H NMR (300.13 MHz, DMSO-d_6): δ 10.32 (s, 1H, O*H*), 8.36 (d, $^3J_{H,H}$= 8.1, 2H, O_2N-C-C*H*), 7.99 (d, $^3J_{H,H}$= 8.4, 2H, N-C-C-C*H*), (s, 2H, N*H₂*) (49).

2-pyridylamidoxime

Picolinonitrile (416 mg, 4.0 mmol, 1.0 eq) was dissolved in EtOH (15 mL) to give a colourless solution. Hydroxylamine hydrochloride (417 mg, 6.0 mmol, 1.5 eq) and NaOH (244 mg, 6.0 mmol, 1.5 eq) were added, and the mixture was then refluxed for 2.5h. The resulting suspension was filtered and the colourless filtrate was evaporated to provide crude 2-pyridylamidoxime as a white powder (406 mg, 85 mol% pure (^{1}H NMR), contaminant: NH_4Cl), which was used without further purification (corrected yield: 2.77 mmol, 69%). ^{1}H NMR (300.13 MHz, DMSO-d_6): δ 10.04 (s, 1H, O*H*), 8.58 (d, $^3J_{H,H}$= 4.5, 1H, Ar-*H*), 7.89-7.80 (m, 2H, Ar-*H*), 7.44 (d, $^3J_{H,H}$= 5.2, 1H, Ar-*H*), 6.12 (br. s, 2H, NH_2) (50).

Syntheses 36.0-36.12

5-phenethyl-3-phenyl -1,2,4-oxadiazole (36.0)

Benzamidoxime (272 mg, 2.0 mmol, 1.0 eq) was dissolved in toluene (5 mL) and cooled to 0 °C, before NEt_3 (0.28 mL, 2.0 mmol, 1.0 eq) and hydrocinnamoyl chloride (0.30 mL, 2.0 mmol, 1.0 eq) were added dropwise. The resulting orange suspension was stirred for 2h at 0 °C, during which it turned lighter. The resulting suspension was refluxed for 5h. The obtained orange/brown mixture was evaporated. The resulting solid was dissolved in DCM (5 mL), and washed with water (3x 5 mL), brine (1x 5 mL), a saturated aqueous Na_2CO_3 solution (3x 5 mL) and brine again (1x 5 mL). Evaporation provided 5-phenethyl-3-phenyl-1,2,4-oxadiazole as a brown oil (255 mg, 1.02 mmol, 51%). ^{1}H NMR (600.13 MHz, $CDCl_3$): δ 8.07 (dd, $^3J_{H,H}$= 7.6, 2H, Ar-*H*), 7.55-7.50 (m, 3H, Ar-*H*), 7.34-7.24 (m, 5H, Ar-*H*), 3.29 (t, $^3J_{H,H}$= 5.5, 2H, N-C-C*H₂*), 3.24 (t, $^3J_{H,H}$= 5.2, 2H, N-C-CH_2-CH_2). ^{13}C {^{1}H} NMR (150.90 MHz, $CDCl_3$): δ 179.1 (s, N-*C*-O), 168.4 (s, N-*C*-N), 139.5 (s, CH_2-*C*-CH), 131.2 (s, Ar-*C*), 128.9 (s, Ar-*C*), 128.8 (s, Ar-*C*), 128.4 (s, Ar-*C*), 127.5 (s, Ar-*C*), 127.0 (s, N-C-*C*), 127.8 (s, Ar-*C*), 32.7 (s, N-C-CH_2-*C*H_2), 28.6 (s, N-C-*C*H_2). HRMS (ESI-Q-TOF): calcd. for $[C_{16}H_{15}N_2O)]^+$: 251.1179; found: 251.1168.

3-(3-nitrophenyl)-5-phenethyl-1,2,4-oxadiazole (36.1)

Powdered 4Å molecular sieves were added to toluene (5 mL), followed by 3-nitro-phenylamidoxime (181 mg, 1.0 mmol, 1.0 eq) and Na_2CO_3 (106 mg, 1.0 mmol, 1.0 eq). The suspension was cooled to 0 °C and hydrocinnamoyl chloride (0.15 mL, 1.0 mmol, 1.0 eq) was added dropwise. After stirring for 30 min at 0 °C, the resulting yellow suspension

was refluxed for 17h. The obtained orange suspension was filtered. Evaporation of the yellow filtrate provided 3-(3-nitrophenyl)-5-phenethyl-1,2,4-oxadiazole as a brown oil (247 mg, 0.84 mmol, 84%). ^{1}H NMR (500.23 MHz, $CDCl_3$): δ 8.91 (s, 1H, O_2N-C(C*H*)-C), 8.39 (d, $^{3}J_{H,H}$= 7.9 Hz, 1H, N-C-C-C*H*), 8.34 (d, $^{3}J_{H,H}$= 8.1 Hz, 1H, O_2N-C-C*H*), 7.67 (t, $^{3}J_{H,H}$= 8.1 Hz, 1H, O_2N-C-CH-C*H*), 7.31 (t, $^{3}J_{H,H}$= 8.5, 2H, CH_2-C-CH-C*H*), 7.25-7.22 (m, 5H, Ar-*H*), 3.31-3.27 (m, 2H, N-C-C*H2*), 3.25-3.19 (m, 2H, N-C-CH_2-C*H2*). ^{13}C {^{1}H} NMR (125.78 MHz, $CDCl_3$): δ 180.0 (s, N-*C*-O), 166.8 (s, N-*C*-N), 148.7 (s, O_2N-*C*), 139.3 (s, CH_2-*C*-CH), 133.1 (s, N-C-C-*C*H), 130.1 (s, O_2N-C-CH-*C*H), 128.8 (s, CH_2-C-CH-*C*H), 128.8 (s, N-C(N)-*C*), 128.3 (s, Ar-*C*), 126.9 (s, Ar-*C*), 125.7 (s, O_2N-C-*C*H), 122.6 (s, O_2N-C-*C*H-C), 32.6 (s, N-C-CH_2-*C*H_2), 28.5 (s, N-C-*C*H_2). HRMS (ESI-Q-TOF): calcd. for [$C_{16}H_{14}N_3O_3$]: 296.1030; found: 296.1035; calcd. for [Na($C_{16}H_{13}N_3O_3$)]: 318.0849; found: 318.0853.

(*E*)-3-(3-nitrophenyl)-5-styryl-1,2,4-oxadiazole (36.2)

3-Nitrobenzamidoxime (54 mg, 0.30 mmol, 1.0 eq) was dissolved in toluene (5 mL). Na_2CO_3 (24 mg, 0.23 mmol, 0.75 eq) was added to the mixture and it was cooled to 0 °C. Cinnamoyl chloride (50 mg, 0.30 mmol, 1.0 eq) was added dropwise and the suspension was stirred at 0 °C for 1h, after which it was refluxed for 16h. The resulting mixture was filtered and evaporation of the yellow filtrate gave (*E*)-3-(3-nitrophenyl)-5-styryl-1,2,4-oxadiazole as a brown oil (59 mg, 0.20 mmol, 67%). ^{1}H NMR (600.13 MHz, $CDCl_3$): δ 9.00 (s, 1H, O_2N-C(C*H*)-C), 8.46 (d, $^{3}J_{H,H}$= 7.5, 1H, N-C-C-C*H*), 8.38 (d, $^{3}J_{H,H}$= 7.7, 1H, O_2N-C-C*H*), 7.94 (d, $^{3}J_{H,H}$= 16.4, 1H, N-C-CH-C*H*), 7.70 (t, $^{3}J_{H,H}$= 8.2, 1H, O_2N-C-CH-C*H*), 7.66-7.62 (m, 2H, Ar-*H*), 7.48-7.42 (m, 3H, Ar-*H*), 7.08 (d, $^{3}J_{H,H}$= 16.4, 1H, N-C-C*H*).

$^{13}C\{^1H\}$ NMR (150.90 MHz, $CDCl_3$): δ 176.2 (s, N-*C*-O), 167.3 (s, N-*C*-N), 148.8 (s, O_2N-*C*), 143.8 (s, N-C-CH-*C*H), 134.3 (s, C-CH-CH-*C*-CH), 133.2 (s, N-C-C-*C*H), 131.0 (s, Ar-*C*), 130.2 (s, O_2N-C-CH-*C*H), 129.3 (s, Ar-*C*), 129.0 (s, N-C(N)-*C*), 128.2 (s, Ar-*C*), 125.8 (s, O_2N-C-*C*H-CH), 122.8 (s, O_2N-C-*C*H-C), 109.9 (s, N-C-*C*H). HRMS (ESI-Q-TOF): calcd. for [$C_{16}H_{12}N_3O_3$]: 294.0873; found: 294.0873; calcd. for [$Na(C_{16}H_{11}N_3O_3)$]: 316.0693; found: 316.0687.

5-benzyl-3-(3-nitrophenyl)-1,2,4-oxadiazole (36.3)

Powdered 4Å molecular sieves were added to toluene (5 mL), followed by 3-nitrobenzamidoxime (181 mg, 1.0 mmol, 1.0 eq) and Na_2CO_3 (106 mg, 1.0 mmol, 1.0 eq). The mixture was cooled to 0 °C and 2-phenylacetyl chloride (0.13 mL, 1.0 mmol, 1.0 eq) was added dropwise. The obtained yellow suspension was refluxed for 17h. The resulting red suspension was filtered. Evaporation of the red filtrate provided a sticky red solid, which was triturated with pentane, to give 5-benzyl-3-(3-nitrophenyl)-1,2,4-oxadiazole as a red solid (199 mg, 0.71 mmol, 71%). ^1H NMR (500.23 MHz, $CDCl_3$): δ 8.78 (t, $^4J_{H,H}$= 1.8, 1H, O_2N-C(C*H*)-C), 8.26 (dt, $^3J_{H,H}$= 7.7, $^4J_{H,H}$= 1.3, 1H, O_2N-C-C*H*-CH), 8.20 (ddd, $^3J_{H,H}$= 8.1, $^4J_{H,H}$= 2.3, $^4J_{H,H}$= 1.1, 1H, N-(CN)-C-C*H*-CH), 7.52 (t, $^3J_{H,H}$= 8.0, 1H, O_2N-C-CH-C*H*), 7.27-7.20 (m, 5H, Ar-*H*), 4.19 (s, 2H, CH_2). $^{13}C\{^1H\}$ NMR (125.78 MHz, $CDCl_3$): δ 179.0 (s, N-*C*-O), 167.0 (s, N-*C*-N), 148.6 (s, O_2N-*C*), 133.2 (s, CH_2-*C*-CH), 133.1 (s, O_2N-C-*C*H-CH), 130.1 (s, O_2N-C-CH-*C*H), 129.1 (s, Ar-*C*), 129.0 (s, Ar-*C*), 128.7 (s, N-(CN)-*C*), 127.9 (s, Ar-*C*), 125.7 (s, N-(CN)-C-*C*H-CH), 122.6 (s, O_2N-C-*C*H-C), 33.1 (s, *C*H_2). HRMS (ESI-Q-TOF): calcd. for [$C_{15}H_{12}N_3O_3$]: 282.0873; found: 282.0875; calcd. for [$Na(C_{15}H_{11}N_3O_3)$]: 304.0693; found: 304.0696.

5-ethyl-3-(3-nitrophenyl)-1,2,4-oxadiazole (36.4)

3-Nitrobenzamidoxime (181 mg, 1.0 mmol, 1.0 eq) was dissolved in toluene (5 mL). Na_2CO_3 (79 mg, 0.75 mmol, 0.75 eq) was added and the mixture was cooled to 0 °C. Propionyl chloride (0.09 mL, 1.0 mmol, 1.0 eq) was added dropwise, resulting in the formation of yellow solids, which partially dissolved when DCM (5 mL) was added to the mixture. After 1h stirring at 0 °C, the resulting suspension was refluxed for 17h. The resulting yellow suspension was filtered and the yellow filtrate was evaporated. The obtained sticky solid was triturated with pentane to provide 5-ethyl-3-(3-nitrophenyl)-1,2,4-oxadiazole as a yellow solid (180 mg, 0.82 mmol, 82%). ^{1}H NMR (600.13 MHz, $CDCl_3$): δ 8.50 (s, 1H, O_2N-C-C*H*-C), 8.30 (d, $^3J_{H,H}$= 8.3, 1H, N-(CN)-C-C*H*-CH), 8.08 (d, $^3J_{H,H}$= 7.7, 1H, O_2N-C-C*H*-CH), 7.60 (t, $^3J_{H,H}$= 7.9, 1H, O_2N-C-CH-C*H*), 2.52 (q, $^3J_{H,H}$= 7.5, 2H, C*H₂*), 1.25 (t, $^3J_{H,H}$= 6.6, 3H, C*H₃*). ^{13}C {^{1}H} NMR (150.90 MHz, $CDCl_3$): δ 172.0 (s, N-*C*-O), 154.2 (s, N-*C*-N), 148.4 (s, O_2N-*C*), 133.1 (s, O_2N-C-*C*H-CH), 133.0 (s, N-(CN)-*C*), 130.0 (s, O_2N-C-CH-*C*H), 125.7 (s, N-(CN)-C-*C*H-CH), 121.8 (s, O_2N-C-*C*H-C), 26.6 (s, *C*H_2), 9.3 (s, *C*H_3). HRMS (ESI-Q-TOF): calcd. for [$C_{10}H_{10}N_3O_3$]: 220.0717; found: 220.0710.

5-(biphenyl-4-yl)-3-(3-nitrophenyl)-1,2,4-oxadiazole (36.5)

3-Nitrobenzamidoxime (181 mg, 1.0 mmol, 1.0 eq) was dissolved in toluene (5 mL). Na_2CO_3 (79 mg, 0.75 mmol, 0.75 eq) was added and biphenyl-4-yl-carbonyl chloride (217 mg, 1.0 mmol, 1.0 eq) was added dropwise at 0 °C. The resulting yellow suspension was

stirred for 1h at 0 °C. Next, the mixture was refluxed for 42h. The resulting thick brown suspension was diluted with toluene (10 mL) and then filtered. Evaporation of the light-yellow filtrate provided 5-(biphenyl-4-yl)-3-(3-nitrophenyl)-1,2,4-oxadiazole as a thermally sensitive yellow solid (221 mg, 0.64 mmol, 64%). ^{1}H NMR (300.13 MHz, $CDCl_3$): δ 9.07 (s, 1H, O_2N-C-C*H*-C), 8.53 (d, $^{3}J_{H,H}$= 7.5, 1H, O_2N-C-C*H*-CH), 8.40 (d, $^{3}J_{H,H}$= 7.8, 1H, N-(CN)-C-C*H*-CH), 8.31 (d, $^{3}J_{H,H}$= 8.2, 2H, O-C-C-C*H*), 7.80 (d, $^{3}J_{H,H}$= 8.4, 2H, Ar-*H*), 7.73 (t, $^{3}J_{H,H}$= 8.1, 1H, O_2N-C-CH-C*H*), 7.67 (d, $^{3}J_{H,H}$= 7.2, 2H, Ar-*H*), 7.55-7.39 (m, 3H, C-C-CH-C*H* + C-C-CH-CH-C*H*). ^{13}C {^{1}H} NMR (125.78 MHz, $CDCl_3$): δ 176.4 (s, N-*C*-O), 167.5 (s, N-*C*-N), 148.7 (s, O_2N-*C*), 146.0 (s, Ar-*C*), 139.6 (s, Ar-*C*), 137.7 (s, Ar-*C*H), 133.2 (s, Ar-*C*H), 130.2 (s, Ar-*C*H), 129.2 (s, Ar-*C*H), 128.9 (s, C-*C*-CH), 128.9 (s, Ar-*C*H), 128.6 (s, Ar-*C*H), 127.9 (s, Ar-*C*H), 127.3 (s, C-C-CH-*C*H), 125.8 (s, Ar-*C*H), 122.8 (s, O_2N-C-*C*H-C), 122.6 (s, N-(CN)-*C*). HRMS (ESI-Q-TOF): calcd. for [$C_{20}H_{14}N_3O_3$]: 344.1030; found: 344.1029; calcd. for [Na($C_{20}H_{13}N_3O_3$)]: 366.0849; found: 366.0853.

3-(3-chlorophenyl)-5-phenethyl-1,2,4-oxadiazole (36.6)

3-Chlorobenzamidoxime (121 mg, 0.71 mmol, 1.0 eq) was dissolved in toluene (5 mL). Na_2CO_3 (75 mg, 0.71 mmol, 1.0 eq) was added and the beige mixture was cooled to 0 °C. Hydrocinnamoyl chloride (0.11 mL, 0.71 mmol, 1.0 eq) was added dropwise. The mixture was stirred for 30 minutes at 0 °C and subsequently refluxed for 41h. The obtained yellow suspension was filtered and the filtrate was evaporated. The obtained oil was purified by column chromatography (1:19 EtOAc: cyclohexane, 1% formic acid) to give crude 3-(3-chlorophenyl)-5-phenethyl-1,2,4-oxadiazole as a brown oil (41 mg, 83 mol% pure (^{1}H NMR), contaminant: hydrocinnamic acid), which was used without further

purification (corrected yield: 0.13 mmol, 18%). ^{1}H NMR (600.13 MHz, $CDCl_3$): δ 8.09 (t, $^4J_{H,H}$= 1.8, 1H, Cl-C-C*H*-C), 7.97 (dt, $^3J_{H,H}$= 7.7, $^4J_{H,H}$= 1.3, 1H, Cl-C-C*H*-CH), 7.48 (ddd, $^3J_{H,H}$= 8.0, $^4J_{H,H}$= 2.2, $^4J_{H,H}$= 1.1, 1H, N-(CN)-C-C*H*-CH), 7.42 (t, $^3J_{H,H}$= 7.8, 1H, Cl-C-CH-C*H*) , 7.25-7.16 (m, 5H, Ar-*H*), 3.29-3.24 (m, 2H, N-C-CH_2-CH_2), 3.23-3.19 (m, 2H, N-C-CH_2). ^{13}C {^{1}H} NMR (150.90 MHz, $CDCl_3$): δ 179.5 (s, N-*C*-O), 167.5 (s, Cl-*C*), 139.4 (s, CH_2-CH_2-*C*-CH), 135.1 (s, N-*C*-N), 131.3 (s, Cl-C-*C*H), 130.3 (s, Cl-C-CH-*C*H), 128.9 (s, Ar-*C*), 128.7 (s, N-(CN)-*C*), 128.4 (s, Ar-*C*), 127.7 (s, N-(CN)-C-*C*H-CH), 126.9 (s, Ar-*C*), 125.6 (s, Cl-C-*C*H-CH), 32.7 (s, N-C-*C*H_2), 28.6 (s, N-C-CH_2-*C*H_2). HRMS (ESI-Q-TOF): calcd. for [$C_{16}H_{14}ClN_2O$]: 285.0789; found: 285.0789; calcd. for [Na($C_{16}H_{13}ClN_2O$)]: 307.0609; found: 307.0609.

3-(4-nitrophenyl)-5-phenethyl-1,2,4-oxadiazole (36.7)

Powdered 4Å molecular sieves were added to toluene (5 mL), followed by 4-nitrobenzamidoxime (80 mg, 0.44 mmol, 1.0 eq) and Na_2CO_3 (47 mg, 0.44 mmol, 1.0 eq). The suspension was cooled to 0 °C and hydrocinnamoyl chloride (0.07 mL, 0.44 mmol, 1.0 eq) was added dropwise. The mixture was stirred for 30 minutes at 0 °C and subsequently refluxed for 17h. The resulting brown suspension was filtered and the orange filtrate was evaporated. The resulting solid was triturated with pentane to provide crude 3-(4-nitrophenyl)-5-phenethyl-1,2,4-oxadiazole as an orange solid (92 mg, 67 mol% pure (^{1}H NMR), contaminant: hydrocinnamic acid), which was used without further purification (corrected yield: 0.25 mmol, 57%). ^{1}H NMR (500.23 MHz, $CDCl_3$): δ 8.34 (d, $^3J_{H,H}$= 8.8, 2H, O_2N-C-C*H*), 8.27 (d, $^3J_{H,H}$= 8.8, 2H, O_2N-C-CH-C*H*), 7.35-7.18 (m, 5H, Ar-*H*), 3.33-3.27 (m, 2H, N-C-CH_2-CH_2), 3.25-3.20 (m, 2H, N-C-CH_2). ^{13}C {^{1}H} NMR (125.78

MHz, $CDCl_3$): δ 174.8 (s, N-*C*-O), 166.9 (s, N-*C*-N), 149.5 (s, O_2N-*C*), 139.3 (s, CH_2-*C*H_2-C), 132.9 (s, N-(CN)-*C*), 128.9 (s, Ar-*C*), 128.5 (s, Ar-*C*), 128.4 (s, N-(CN)-C-*C*H), 127.0 (s, Ar-*C*), 124.2 (s, O_2N-C-*C*H), 32.6 (s, N-C-*C*H_2), 28.6 (s, N-C-CH_2-*C*H_2). HRMS (ESI-Q-TOF): calcd. for [$C_{16}H_{14}N_3O_3$]: 296.1030; found: 296.1044; calcd. for [Na($C_{16}H_{13}N_3O_3$)]: 318.0849; found: 318.0838.

5-phenethyl-3-(2-pyridyl)-1,2,4-oxadiazole (36.8)

2-Pyridylamidoxime (137 mg, 1.0 mmol, 1.0 eq) was added to toluene (5 mL), followed by Na_2CO_3 (106 mg, 1.0 mmol, 1.0 eq), and the resulting beige suspension was cooled to 0 °C. Hydrocinnamoyl chloride (0.15 mL, 1.0 mmol, 1.0 eq) was added dropwise and the yellow suspension was refluxed for 58h. After 2 hours additional toluene (10 mL) was added. The brown suspension was filtered and the filtrate was evaporated. After a filtration over silica (3:7 EtOAc : cyclohexane, 1% NEt_3), 5-phenethyl-3-(pyridine-2-yl)-1,2,4-oxadiazole was obtained as a brown oil (77 mg, 0.31 mmol, 31%). ^{1}H NMR (600.13 MHz, $CDCl_3$): δ 8.81 (d, $^3J_{H,H}$= 5.1, 1H, N-C*H*-C), 8.13 (d, $^3J_{H,H}$= 7.9, 1H, N-C*H*-CH-CH-C), 7.85 (td, $^3J_{H,H}$= 7.9, $^4J_{H,H}$= 1.8, 1H, N-CH-C*H*-CH-C), 7.43 (ddd, $^3J_{H,H}$= 7.7, $^3J_{H,H}$= 4.7, $^4J_{H,H}$= 1.1, 1H, N-CH-CH-C*H*-C), 7.32 (t, $^3J_{H,H}$= 7.6, 2H, CH_2-C-CH-C*H*), 7.29-7.20 (m, 3H, Ar-*H*), 3.33-3.28 (m, 2H, N-C-CH_2-C*H₂*), 3.27-3.22 (m, 2H, N-C-C*H₂*). ^{13}C {^{1}H} NMR (150.90 MHz, $CDCl_3$): δ 179.9 (s, N-*C*-O), 168.2 (s, N-*C*-N), 150.5 (s, N-*C*H-C), 146.4 (s, N-(CN)-*C*), 139.4 (s, CH_2-*C*-CH), 137.1 (s, N-CH-*C*H-CH-C), 128.8 (s, CH_2-C-CH-*C*H), 128.3 (s, Ar-*C*), 126.8 (s, Ar-*C*), 125.5 (s, N-CH-CH-*C*H-C), 123.1 (s, N-*C*H-CH-CH-C), 32.7 (s, N-C-*C*H_2), 28.7 (s, N-C-CH_2-*C*H_2). HRMS (ESI-Q-TOF): calcd. for [$C_{15}H_{14}N_3O$]: 252.1131; found: 252.1128; calcd. for [Na($C_{15}H_{13}N_3O$)]: 274.0951; found: 274.0949.

N-(benzimidoyl)-3-phenylpropanamide (36.9)

Under N_2 atmosphere, benzimidamide hydrochloride (211 mg, 1.35 mmol, 1.0 eq) was dissolved in MeOH (10 mL), to which NaOMe (25% in MeOH, 0.31 mL, 1.35 mmol, 1.0 eq) and hydrocinnamoyl chloride (0.20 mL, 1.35 mmol, 1.0 eq) were added. The mixture was stirred for 2 days, after which additional NaOMe (25% in MeOH, 0.31 mL, 1.35 mmol, 1.0 eq) was added. The mixture was stirred again for 2 days. Under atmospheric conditions, the mixture was evaporated and the resulting substance was extracted into DCM. The DCM fraction was washed with water (3x 5 mL) and brine (1x 5 mL) and evaporated. The resulting oil was filtered over silica (4:6 EtOAc : cyclohexane, 1% NEt_3), and dried *in vacuo* to provide *N*-(benzimidoyl)-3-phenylpropanamide as a colourless oil (44 mg, 0.17 mmol, 13%). ^{1}H NMR (600.13 MHz, $CDCl_3$): δ 7.84 (d, $^3J_{H,H}$= 6.6, 2H, NH-C-C-C*H*), 7.54 (t, $^3J_{H,H}$= 7.1, 1H, NH-C-C-CH-CH-C*H*), 7.46 (t, $^3J_{H,H}$= 7.3, 2H, NH-C-C-C*H*), 7.32-7.24 (m, 4H, Ar-*H*), 7.23-7.17 (m, 1H, Ar-*H*), 3.06 (t, $^3J_{H,H}$= 7.5 , 2H, NH-C-CH_2), 2.90 (t, $^3J_{H,H}$= 8.2, 2H, NH-C-CH_2-CH_2). ^{13}C {^{1}H} NMR (150.90 MHz, $CDCl_3$): δ 179.1 (s, *C*=O), 171.4 (s, *C*=NH), 141.8 (s, CH_2-*C*-CH), 135.2 (s, NH=C-*C*), 132.3 (s, NH-C-C-CH-CH-*C*H), 129.0 (s, NH-C-C-CH-*C*H), 128.5 (s, Ar-*C*), 127.3 (s, NH-C-*C*H), 126.0 (s, Ar-*C*), 42.8 (s, O=C-*C*H_2), 31.7 (s, O=C-CH_2-*C*H_2). HRMS (ESI-Q-TOF): calcd. for [Na($C_{16}H_{16}N_2O$)]: 275.1155; found: 275.1143.

N-benzyl-3-phenylpropanamide (36.10)

To a colorless solution of benzylamine (0.55 mL, 5.0 mmol, 1.0 eq) and NEt_3 (0.84 mL, 6.0 mmol, 1.2 eq) in DCM (100 mL), 3-phenylpropanoyl chloride (0.74 mL, 5.0 mmol, 1.0 eq) was added dropwise at 0 °C. The resulting solution was stirred for 18h at RT, after which it was washed with water (3 x 100 mL) and brine (1 x 100 mL). Evaporation and drying *in vacuo* provided *N*-benzyl-3-phenylpropanamide as a yellow oil (1.20 g, 5.0 mmol, quant.). ^1H NMR (300.13 MHz, $CDCl_3$): δ 7.30-7.04 (m, 10H, Ar-*H*), 5.62 (br. s, 1H, N*H*), 4.35 (d, $^3J_{H,H}$=5.7 Hz, 2H, NH-C*H₂*), 2.95 (t, $^3J_{H,H}$=7.5 Hz, 2H, (CO)-CH_2), 2.46 (t, $^3J_{H,H}$=7.5 Hz, 2H, (CO)-CH_2-CH_2) (51).

_N_-(3-phenylpropyl)benzamide (36.11)

To a colorless solution of 3-phenylpropan-1-amine (0.71 mL, 5.0 mmol, 1.0 eq) and NEt_3 (0.84 mL, 6.0 mmol, 1.2 eq) in DCM (50 mL), benzoyl chloride (0.58 mL, 5,0 mmol, 1.0 eq) was added dropwise at 0 °C. The resulting solution was stirred for 1 day at RT, after which it was washed with water (3 x 50 mL) and brine (1 x 50 mL). Evaporation and drying *in vacuo* provided *N*-(3-phenylpropyl)benzamide (1.20 g, 5.0 mmol, quant.). ^1H NMR (500.23 MHz, $CDCl_3$): δ 7.68-7.64 (m, 2H, Ar-*H*), 7.50-7.46 (m, 1H, Ar-*H*), 7.43-7.39 (m, 2H, Ar-*H*), 7.32-7.28 (m, 2H, Ar-*H*), 7.24-7.20 (m, 3H, Ar-*H*), 6.06 (br. s, 1H, N*H*), 3.51 (dd, $^3J_{H,H}$=14.1, $^3J_{H,H}$=6.9, 2H, NH-CH_2), 2.74 (t, $^3J_{H,H}$=7.7, 2H, CH_2-C), 1.97 (q, $^3J_{H,H}$=7.3, 2H, NH-CH_2-CH_2) (52).

3,5-diphenyl-1,2,4-oxadiazole (36.12)

Powdered 4Å molecular sieves were added to toluene (10 mL), followed by benzamidoxime (272 mg, 2.0 mmol, 1.0 eq) and Na_2CO_3 (219 mg, 2.1 mmol, 1.0 eq). The dark yellow suspension was cooled to 0 °C and benzoyl chloride (0.23 mL, 2.0 mmol, 1.0 eq) was added dropwise. The mixture was stirred for 10 minutes at 0 °C and subsequently refluxed for 3 days. The resulting yellow suspension was filtered and the filtrate was evaporated to give 3,5-diphenyl-1,2,4-oxadiazole as a yellow powder (388 mg, 1.8 mmol, 87% yield). ^{1}H NMR (300.13 MHz, $CDCl_3$): δ 8.27-8.14 (m, 4H, Ar-*H*), 7.66-7.44 (m, 6H, Ar-*H*) (53).

Supplementary Figures

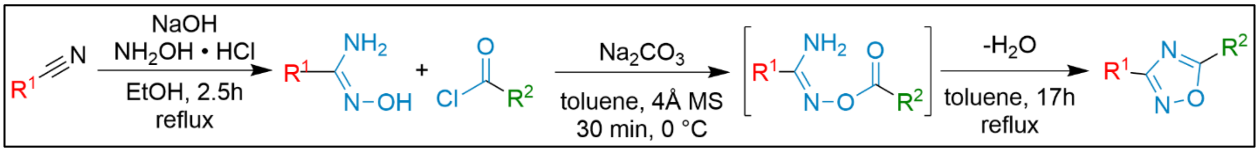

Figure S1. Optimized synthesis route towards 1,2,4-oxadiazoles 36.0-36.8 and 36.12.

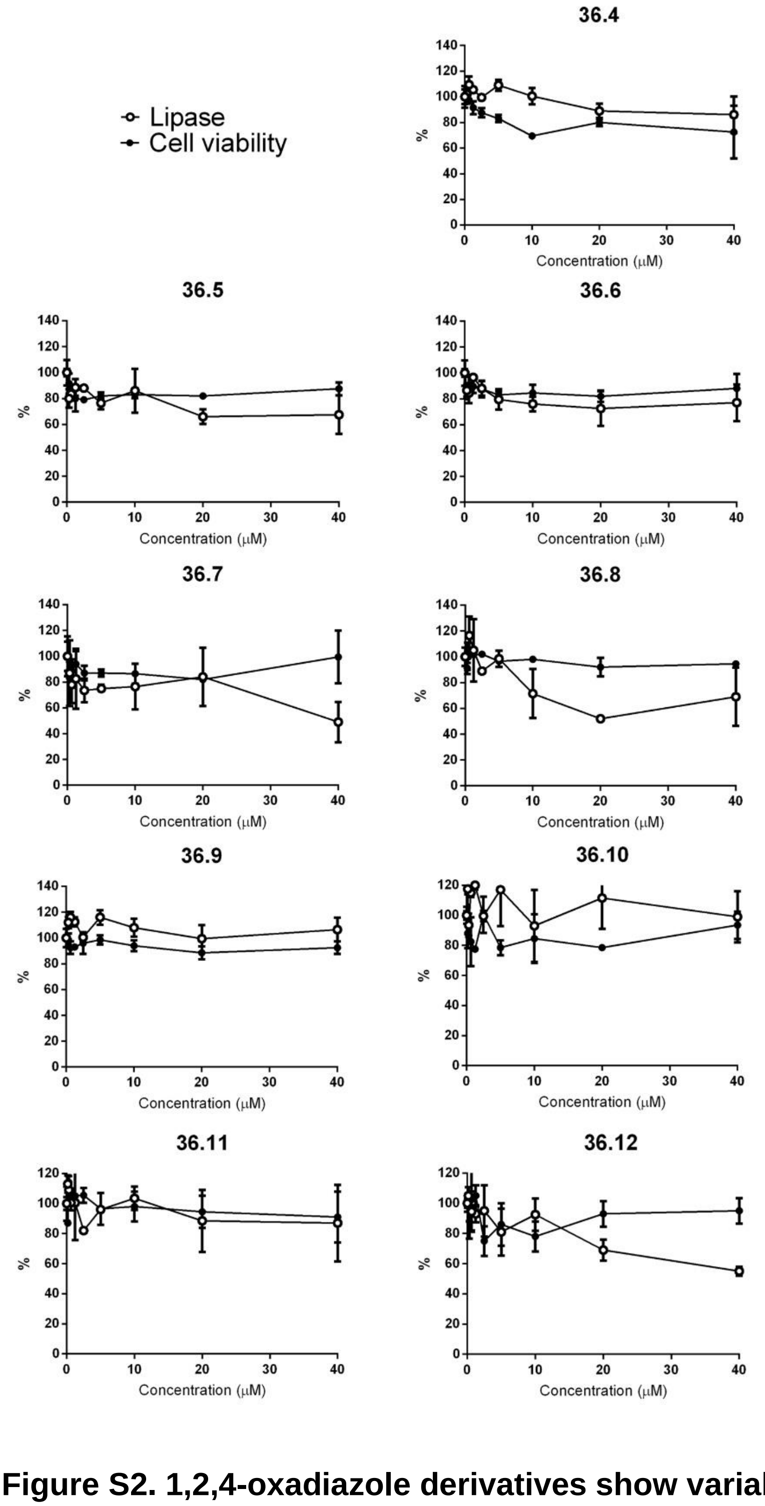

Figure S2. 1,2,4-oxadiazole derivatives show variable inhibition of LipY activity. *M. marinum* carrying pSMT3-lipY-mspA was grown in the presence of test compounds. After three days, lipase activity was measured using DGGR as a substrate. Subsequently, cell viability was determined using REMA.

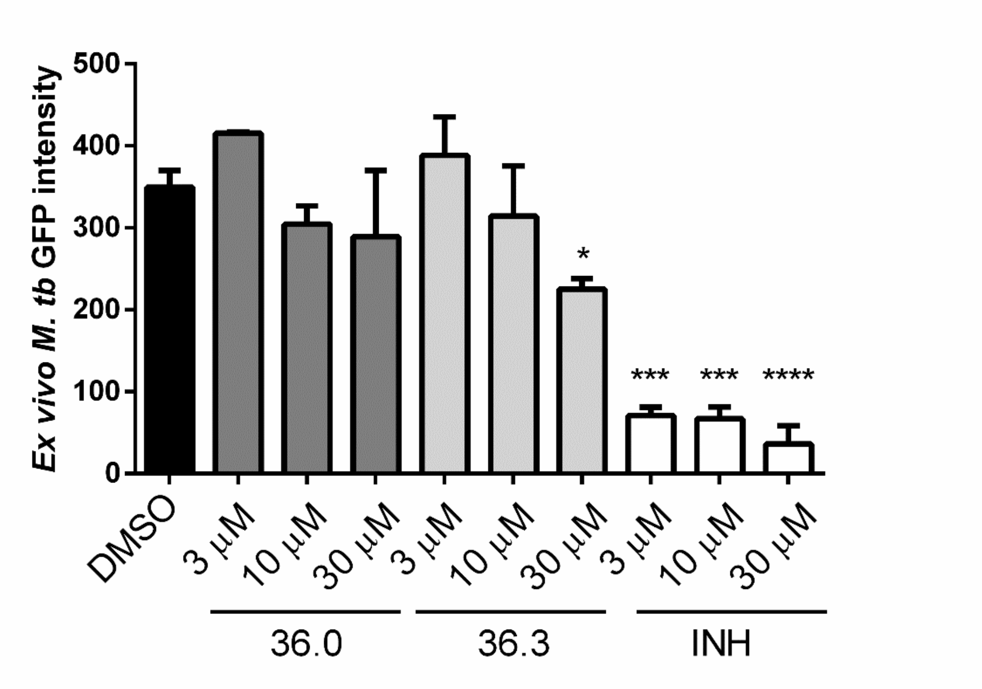

Figure S3: Compound 36.3 significantly reduces bacterial burden in *M. tuberculosis*-infected macrophages. Differentiated THP-1 macrophage cells were infected with *M. tuberculosis* harboring pTetDuo vector (having GFP under a tetracycline-inducible promoter). Cells were treated with test compounds or isoniazid (INH) for 6 days. Subsequently, ATc (anhydrotetracycline) was added to induce the expression of GFP in *M. tuberculosis*. Finally, cells were stained with Hoechst dye to detect macrophages and GFP signals representing viable bacteria were scored. DMSO and INH were used as a negative control or positive control. Statistical significance was determined by one-way ANOVA with Dunnett post-hoc test by comparing the signal from the DMSO-treated control sample with each treatment group (*$p \leq 0.05$, ***$p \leq 0.001$, ****$p \leq 0.0001$).

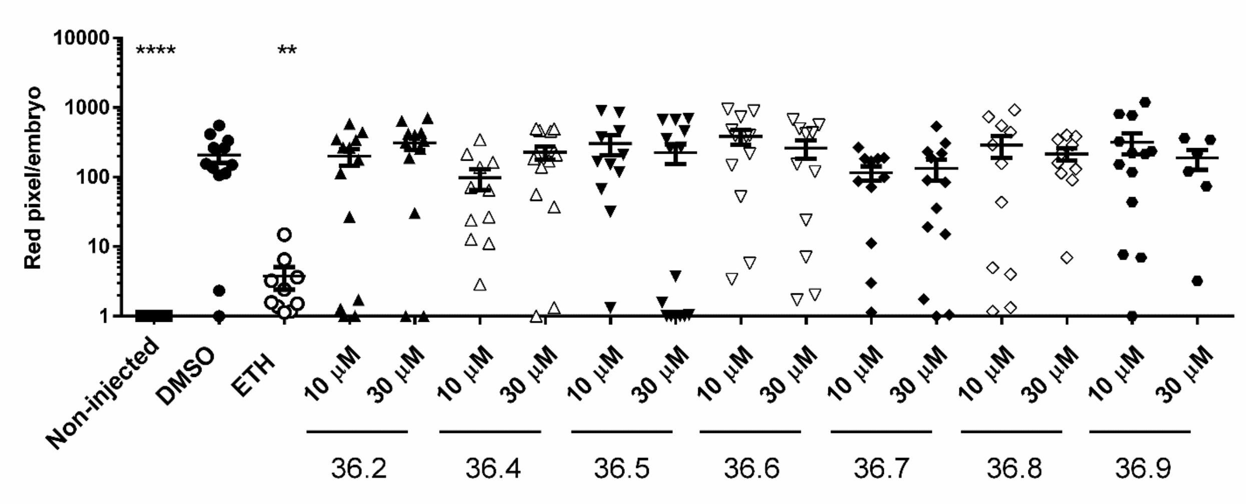

Figure S4. 1,2,4-oxadiazole derivatives that do not show activity in the zebrafish infection model. Zebrafish were infected via yolk injection with *M. marinum* (transformed with pMS2-tdTomato) and treated with tested compounds at 10 µM or 30 µM concentrations with 12 embryos per group. At 3-day post-treatment, the infection was quantified by determining the fluorescent intensity of Tdtomato. Deformed or dead fish were removed from the analysis. Viable embryos were scored, with each dot representing a single embryo. Statistical significance was determined by one-way ANOVA with Dunnett post-hoc test by comparing the signal from the DMSO-treated control with each test group (***p* ≤0.01, *****p* ≤0.0001).

Strain	Parental strain	References
E. coli DH5α	*recA1*; *endA1*; *gyrA96*; *thi*; *rel*A1; *hsd*R17(r_{K-},m_{K+}); *sup*E44; φ 80Δ *lacZ* Δ M15; Δ *lacZ*(YA-argF)UE169	(54)
M. marinum M[USA]	Laboratory strain	(20)
M. tuberculosis H37Rv	Laboratory strain	ATCC 25618

Table S1: Bacterial strains used in the study

Plasmid names	Features	Source
pMN016	p_{smyc}-*mspA*; oriE(ColE1); pAL5000 origin; *hyg*$_R$; 6164 bp	(55)
pSMT3-LipY-MspA	p_{smyc}-*mspA*-*lipYtb*; oriE(ColE1); pAL5000 origin; *aph*; *kan*$_R$; 7633 bp	(25)
pMS2-tdTomato	p_{wmyc}-*tdTomato*; oriE(ColE1); pAL5000 origin; *hyg*$_R$; 6132 bp	(24)
pTetDuo	p_{smyc}-*tdtomato; tetR*$_{on}$,p_{teto}-*mgfp2+,*oriE (ColE1), pAL5000 origin; *hyg*$_{R;}$ 7936bp	(23)
pML2424	pUC origin; pAL5000ts; *sacR*; *sacB*; p_{wmyc}-*tdTomato*; *loxP*-p_{imyc}-*mycgfp2+-hyg-loxP*; 9527 bp	(56)
p-pro$_{mmar_5294}$-tdTomato	p_{mmar_5294}-*tdTomato*; oriE(ColE1); pAL5000 origin; *hyg*$_R$; 6380 bp	This study

Table S2: Plasmids used in the study

Primers	Sequences
FW-pro-mmar5294	taatactgtttaaactctagcggctaaaaccgtccgaggg
RV-pro-mmar5294	tcctcgcccttgctcaccatcacaccactcctctgcgata
FW-Tdtomato	tatcgcagaggagtggtgtgatggtgagcaagggcgagga
RV-Tdtomato	ccaattaattagctaaagctttacttgtacagctcgtcca

Table S3: Primers used in the study
